# USP18 is crucial for IFN-γ-mediated inhibition of B16 melanoma tumorigenesis and antitumor immunity

**DOI:** 10.1186/1476-4598-13-132

**Published:** 2014-05-31

**Authors:** Bangxing Hong, Haiyan Li, Yong Lu, Mingjun Zhang, Yuhuan Zheng, Jianfei Qian, Qing Yi

**Affiliations:** 1 Department of Cancer Biology, Lerner Research Institute, Cleveland Clinic, 9500 Euclid Avenue/NB40, Cleveland, OH 44195, USA

**Keywords:** USP18, Immunosurveillance, Immunotherapy

## Abstract

**Background:**

Interferon (IFN)-γ-mediated immune response plays an important role in tumor immunosurveillance. However, the regulation of IFN-γ-mediated tumorigenesis and immune response remains elusive. USP18, an interferon stimulating response element, regulates IFN-α-mediated signaling in anti-viral immune response, but its role in IFN-γ-mediated tumorigenesis and anti-tumor immune response is unknown.

**Method:**

In this study, USP18 in tumorigenesis and anti-tumor immune response was comprehensively appraised *in vivo* by overexpression or downregulation its expression in murine B16 melanoma tumor model in immunocompetent and immunodeficient mice.

**Results:**

Ectopic expression or downregulation of USP18 in B16 melanoma tumor cells inhibited or promoted tumorigenesis, respectively, in immunocompetent mice. USP18 expression in B16 melanoma tumor cells regulated IFN-γ-mediated immunoediting, including upregulating MHC class-I expression, reducing tumor cell-mediated inhibition of T cell proliferation and activation, and suppressing PD-1 expression in CD4^+^ and CD8^+^ T cells in tumor-bearing mice. USP18 expression in B16 melanoma tumor cells also enhanced CTL activity during adoptive immunotherapy by prolonging the persistence and enhancing the activity of adoptively transferred CTLs and by reducing CTL exhaustion in the tumor microenvironment. Mechanistic studies demonstrated that USP18 suppressed tumor cell-mediated immune inhibition by activating T cells, inhibiting T-cell exhaustion, and reducing dendritic cell tolerance, thus sensitizing tumor cells to immunosurveillance and immunotherapy.

**Conclusion:**

These findings suggest that stimulating USP18 is a feasible approach to induce B16 melanoma specific immune response.

## Introduction

The immune system has developed specific mechanisms to induce tumor immunosurveillance and antitumor immune responses [1-3]. These include activation of innate immune cells, such as NK cells and phagocytes, and the tumor antigen-specific adaptive immune response. Cytotoxic T lymphocytes (CTLs) are the main adaptive immune cells which lyse tumor cells in an antigen-specific manner [4].

Activated NK cells and CTLs secrete various effector molecules to lyse tumor cells. They both secrete the type-II interferon, IFN-γ, to enhance anti-tumor activity, which includes enhancing antigen presentation and promoting the proliferation, expansion and survival of CD8^+^ T cells [5,6]. IFN-γ is a pleiotropic cytokine that has diverse biological functions [7] and binds to cognate receptors at the cell surface and activates the JAK-STAT pathway to induce expression of IFN -stimulated genes (ISGs) [8]. Several mechanisms exist to terminate IFN-γ signaling, including induction of SOCS family protein expression [9,10]. In contrast, the type-I IFN-α/-β can induce ubiquitin-specific protease 18 (USP18) expression to attenuate type-I IFN signaling [11,12]. USP18 regulates type-I IFN signaling through its deubiquitinase activity towards free ISG15 production, but also binds the IFNAR2 receptor to inhibit JAK/STAT activation [12]. Whether USP18 also regulates IFN-γ signaling is still not completely understood.

In this report, we investigated the function of USP18 in IFN-γ signaling in B16 melanoma cells in vitro and in vivo and found that IFN-γ or CTLs activated USP18 expression in tumor cells. Mechanistic studies using immuocompromised mice or immune cells depletion, or antigen-specific CTL immunotherapy showed that USP18 expression in B16 melanoma cells was essential for maintaining tumor antigen-specific CTL activity, persistence, and for IFN-γ signaling-mediated tumor immunesurveillance. This study is not only important for elucidating the regulation of CTL immunotherapy, but also provides a scientific basis for developing novel immunotherapeutic strategies to target USP18 in B16 melanoma cells to induce innate and adaptive immune responses against tumors.

## Materials and methods

### Materials and antibodies

Adenovirus containing mouse USP18 (Ad-mUSP18) was purchased from Applied Biological Materials Inc. (Richmond, BC, Canada). We prepared lentivirus constructs containing mouse USP18 shRNA. Rabbit and goat anti-mouse USP18 antibodies were kindly provided by Dr. Ethan Dmitrovsky (Dartmouth-Hitchcock Medical center, Dartmouth College, USA) or purchased from Santa Cruz Biotechnology.

### Mouse models

C57BL/6, NOD-SCID-IL2Rγ^-/-^ (NSG), *Ifng*^-/-^, OT-1 and OT-2 C57BL/6 and pmel-1 C57BL/6 transgenic mice were purchased from Jackson Laboratory. All mice were 6- to 7 weeks of age at the time of experiment, and at least 5 mice per group were used in each experiment. Mice were housed and experimental procedures were performed in accordance with the IACUC guidelines at University of Texas MD Anderson Cancer Center and Cleveland Clinic.

### Generation of stable USP18 overexpression and knockdown cancer cells

Overexpression of USP18 into the tumor cell line B16 was accomplished by transduction of adenovirus Ad-mUSP18- followed by cell sorting to select GFP-positive tumor cells (B16-USP18, B16-OVA-USP18). Stable knockdown of USP18 was accomplished by lentivirus shUSP18 transduction of B16 and B16-OVA tumor cells and sorting for GFP-positive tumor cells (B16-shUSP18, B16-OVA-shUSP18).

### Subcutaneous and intravenous B16 melanoma models

Subcutaneous and intravenous murine melanoma models were established as described elsewhere [13]. Briefly, for the subcutaneous tumor model, 0.5- to 1.0 million B16-OVA-shCon, B16-OVA-USP18, B16-OVA-shUSP18 tumor cells were subcutaneously inoculated. For the intravenous tumor model, 0.3- to 1.0 million of tumor cells were intravenously injected, and lung tumor foci were counted to assess tumor burdens. In some experiments, the tumor model was first established, followed by adoptive transfer of antigen-specific CTLs to the tumor-bearing mice.

### Flow cytometry and cell sorting

Cells from tumor, spleen, lymph nodes and lung were mechanically dissociated, and the red blood cells were removed by ACK lysis buffer (Lonza). Cells were first blocked with Fc antibody and then labeled with different combinations of antibodies. Data were acquired with an LSR Fortessa flow cytometer (BD Biosciences), and analysis was performed using Flowjo software. Cell sorting was performed using a BD FACSAria cell sorter (BD Biosciences). Fluorochrome conjugated CD4, CD8, CD11b, NK1.1, PD1, NKG2D, CD86, MHC I, MHC II, KLRG1, IFNγ and TNFα were bought from BD Biosciences. PE conjugated OT-1 tetramer were bought from Beckman Coulter, Inc.

### Cell proliferation and apoptosis assays

Cell proliferation was assessed by a BrdU incorporation assay. Cells cultured in vitro were treated with 10 μM BrdU for 30 min or 16 hrs when the cells were in exponential growth. Cells were harvested and stained with BrdU-APC/7AAD. For apoptosis assay, cells were cultured until they reached exponential growth and then were collected and stained with Annexin-V/PI.

### Western blot

Cell lysates, 20 μg, were electrophoresed on 4-12% SDS-PAGE gels, and transferred onto 0.22 μm nitrocellulose membrane (Hybond, Amersham Biosciences, Inc.). The membrane was then incubated with specific antibody followed by horseradish peroxidase-conjugated secondary antibody (Amersham Biosciences, Inc.). The expressed protein was detected with an ECL Plus Western blotting detection kit (Amersham Biosciences, Inc.).

### qRTPCR

mRNA was prepared from cells or tissues using a Qiagen RNAeasy Kit. cDNA was synthesized using an ABI Biosystems cDNA synthesis kit. Quantitative PCR was conducted with SYBR Green Master Mix (Life Technologies) and run in an ABI750 thermal cycler. The primers for USP18 are: 5′-atgcaggacagtcgacagaa-3′ and 5′- tgtcaagtctgtgtccgtga-3′.

### Immunohistochemisty staining

Paraffin-embedded tumor tissues were processed with pH9.0 antigen retrieval buffer. The slides were blocked with 3% H_2_O_2_ and the serum of the species of secondary antibody. The primary antibody was incubated at 4°C overnight. Slides were washed and secondary antibody was incubated for 20 min at room temperature. Slides were washed three times with 1 × PBST and then developed with DAB and counterstained with H&E and mounted for microcopy analysis.

### Coculture of OT-1 and OT-2 cells with B16-OVA-USP18 tumor cells

B16-OVA-GFP or B16-OVA-USP18 tumor cells were cocultured with or without IFN-γ sensitization (5 ng/ml) and subjected to low dose (10 Gy) irradiation. OT-1- and OT-2-naive T cells were cocultured with irradiated tumor cells for 48- to 72 hours. T-cell proliferation was analyzed by a CFSE dilution assay or ^3^H-incorporation assay. IL-2 and IFN-γ secretion from cocultured T cells was analyzed by ELISA.

### Statistical analysis

Groups were compared using a student’s t-test. Differences were considered significant when *p* < 0.05. All data are presented as mean ± SEM.

## Results

### IFN-γ signaling induces USP18 expression in tumor cells during immunosurveillance

IFN signaling plays important roles in cell activity, including cell growth, differentiation, proliferation, and immune regulation. IFN signaling also is critical for immunosurveillance for tumorigenesis and metastasis [5], including surveillance for induction of MHC class-I molecule expression on tumor cells [14]. We first verified that IFN-α/-β or IFN-γ stimulation increased MHC class-I expression on tumor cells (Additional file 1: Figure S1A). Inoculated tumor cells in IFN-γ signaling-deficient (*Ifng*^-/-^) mice had reduced MHC class-I expression (Additional file 1: Figure S1B).

**Figure 1:**
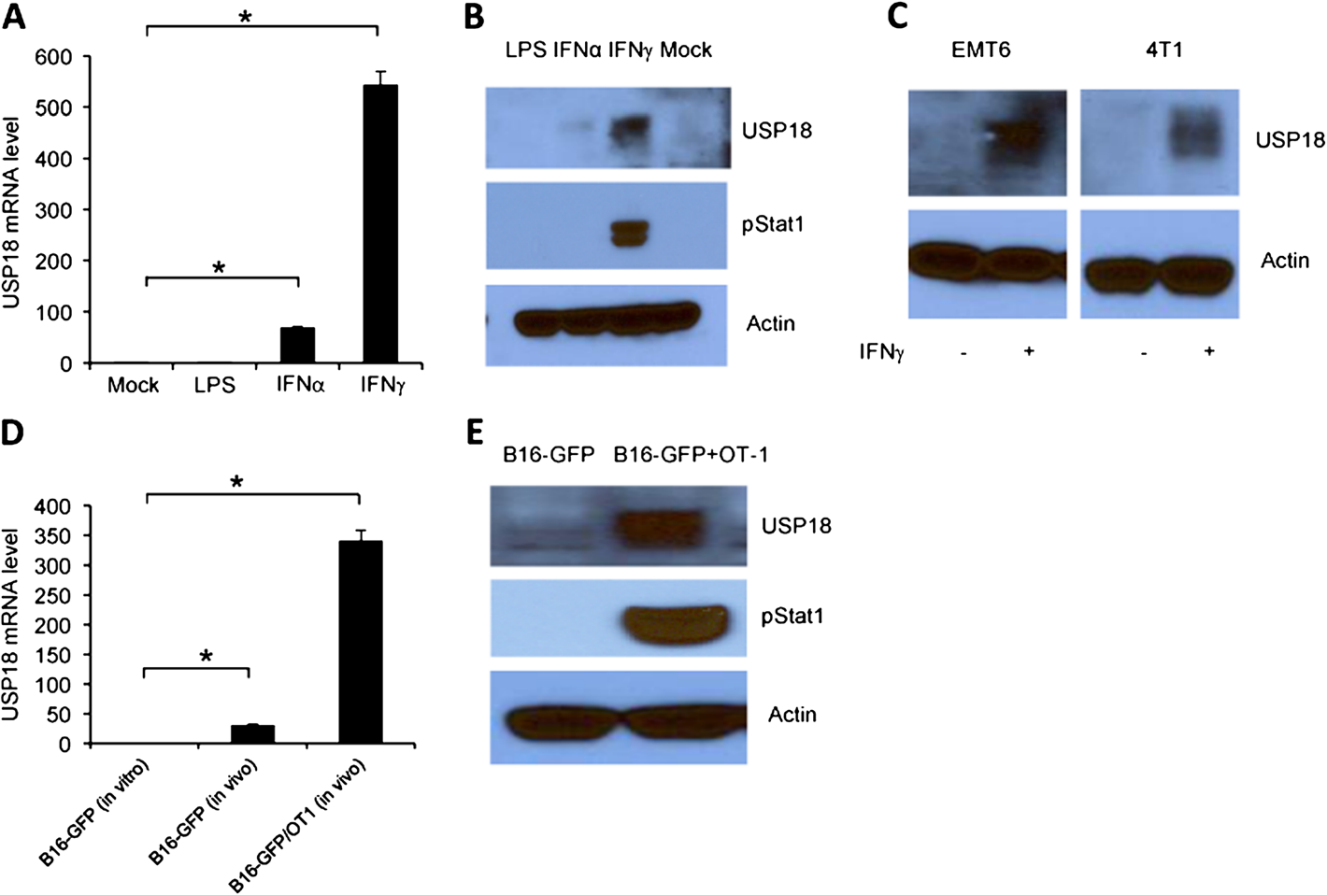
**USP18 expression in tumor cells with IFN-γ signaling.** B16 tumor cells were stimulated in vitro with IFN-γ (10 ng/ml) (R&D systems), IFN-α (10 ng/ml) (R&D systems), or LPS (1 μg/ml) (Invivogen) for 24 hr, then harvested for qRT-PCR assay of USP18 mRNA **(A)** and Western blot assay **(B)** of USP18 and pStat1 protein expression. EMT6 and 4 T1 tumor cells were untreated or treated with IFN-γ (5 ng/ml) for 24 hr, USP18 expression was analyzed by Western blot analysis **(C)**. B16-GFP cells were subcutaneously inoculated into C57BL/6 mice until tumor sizes reached about 5 mm. Some mice received adoptive transfer of 5 × 10^6^ activated OT-1 cells for 48 hr. B16-GFP tumor cells were collected and qRT-PCR analysis of USP18 expression **(D)**. B16 tumor cells were cocultured with activated OT-1 cells for 24 hr and analyzed for USP18 and pStat1 expression **(E)**.

IFN signaling is regulated by various molecules and the canonical factors are the IFN-sensitive response elements (ISREs) [8]. USP18 is an IFN stimulated gene that has been reported to regulate type-I IFN signaling in the anti-viral immune response [12]. To our knowledge, very few studies have investigated USP18 regulation of type-II IFN signaling in tumor immunosurveillance. We assessed USP18 mRNA and protein levels in B16 tumor cells after IFN-α or IFN-γ stimulation and found that IFN-γ was a more potent inducer than IFN-α for USP18 expression (Figure 1A and B), as well as Stat1 phosphorylation, which is IFN-γ signaling downstream gene (Figure 1B). Western blot analysis showed that IFN-γ induced USP18 protein expression in other tumor cell lines such as 4 T1 and EMT6 tumor cells (Figure 1C). To investigate whether endogenous IFN signaling induces USP18 expression during tumor development in vivo, we inoculated C57BL/6 mice with B16-GFP tumor cells. Tumor cell USP18 expression was increased in vivo as compared with in vitro conditions (Figure 1D). To examine exogenous IFN signaling, such as CTL secretion of IFN-γ, we performed adoptive transfer of activated OT-1 cells, which resulted in stronger USP18 expression in tumor cells in vivo (Figure 1D). Similar results were found in in vitro coculture (Figure 1E). These results demonstrated that USP18 was expressed in tumor cells during tumor development regardless of whether IFN signaling was endogenous or exogenous.

### USP18 expression in tumor cells suppresses tumor growth in vivo

**Figure 2:**
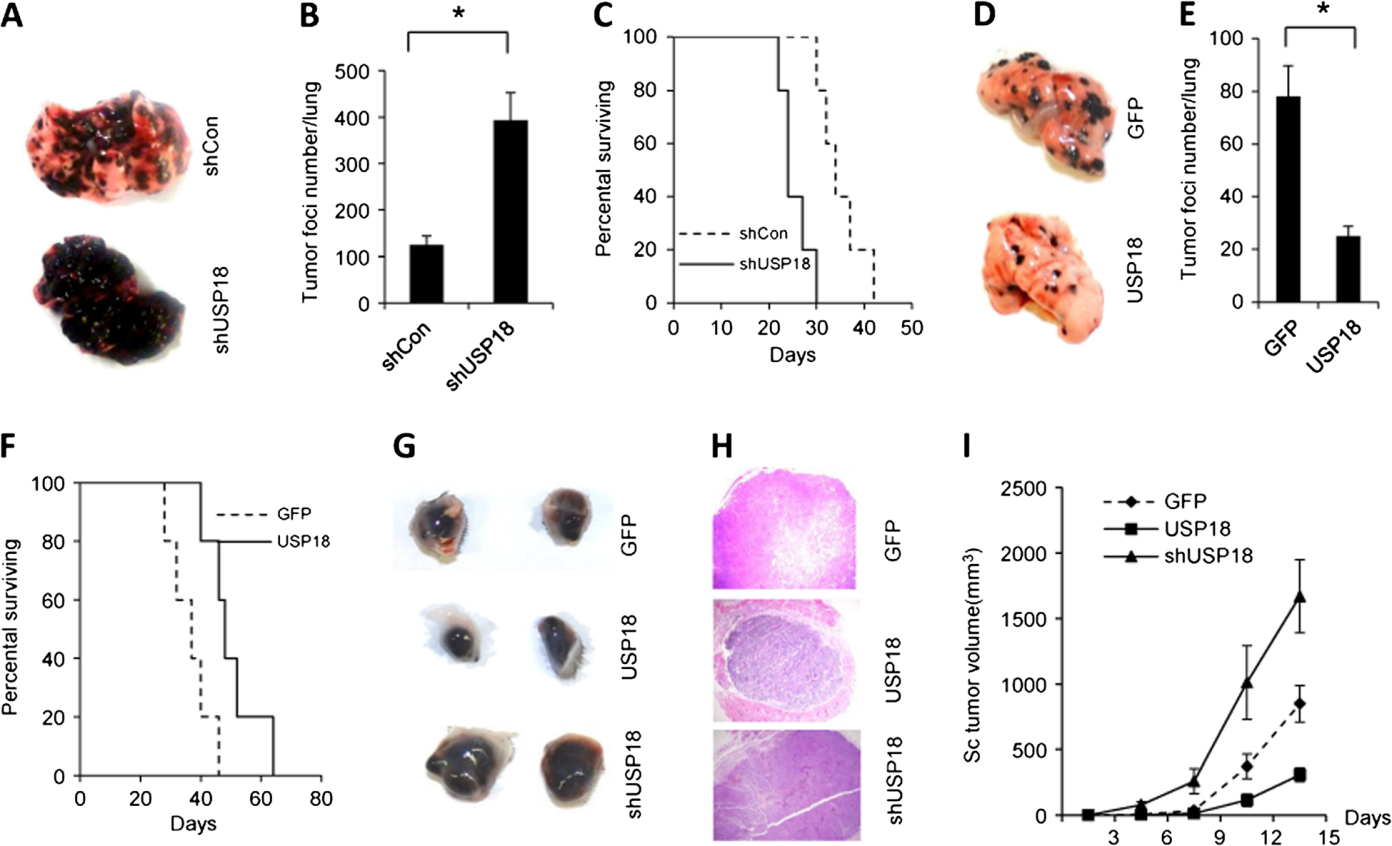
**USP18 expression in tumor cells suppresses tumor growth in vivo.** 3 × 10^5^ B16-OVA-shUSP18 or B16-OVA-shCon tumor cells were intravenously inoculated into C57BL/6 mice. Tumor growth was monitored by counting the lung tumor foci **(A-B)** and the survival rate **(C)**. 2 × 10^5^ B16-OVA-USP18 or B16-OVA-GFP tumor cells were intravenously inoculated into C57BL/6 mice. Tumor growth was monitored by counting the lung tumor foci **(D-E)**, and mouse survival rate over time **(F)**. 1 × 10^6^ B16-OVA-USP18 or B16-OVA-GFP, or B16-OVA-shUSP18 tumor cells were subcutaneously inoculated into C57BL/6 mice. The tumor growth was monitored as tumor volume and by H&E staining **(G-I)**.

To determine whether tumor cell USP18 expression contributes to tumorigenesis in vivo, we stably silenced or ectopically expressed USP18 in B16-OVA tumor cells (Additional file 1: Figure S2A and B). As IFN signaling was required for USP18 expression in tumor cells, knockdown of USP18 was confirmed after IFN-γ stimulation (Additional file 1: Figure S2A). We found that USP18 knockdown or overexpression did not affect the proliferation or apoptosis status of the cells, either with or without IFN-γ stimulation (Additional file 1: Figure S2C and D). B16-OVA-shCon (control), B16-OVA-shUSP18 (USP18 knockdown), or B16-OVA-USP18 (USP18 overexpression) cells were then intravenously inoculated into immunocompetent C57BL/6 mice. Knockdown of USP18 expression significantly increased tumor burden in C57BL/6 mice (Figure 2A and B, and Additional file 1: Figure S3) and shortened mouse survival time (Figure 2C). Conversely, overexpression of USP18 in B16-OVA tumor cells significantly reduced tumor burden in C57BL/6 mice (Figure 2D and E, and Additional file 1: Figure S3) and prolonged mouse survival time (Figure 2F). As tumor cells were intravenously injected into mice, the change in tumor burden was not due to the difference of invasion or migration of tumor cells (Additional file 1: Figure S4A-C).In order to confirm this finding, we subcutaneously inoculated B16-OVA-shCon, B16-OVA-shUSP18, or B16-OVA-USP18 tumor cells into C57BL/6 mice. Tumor growth in mice receiving B16-OVA-USP18 cells was significantly inhibited, whereas tumor growth was enhanced in those receiving B16-OVA-shUSP18 tumor cells (Figure 2G-I).

### USP18 function in tumor growth depends on the immune system

**Figure 3:**
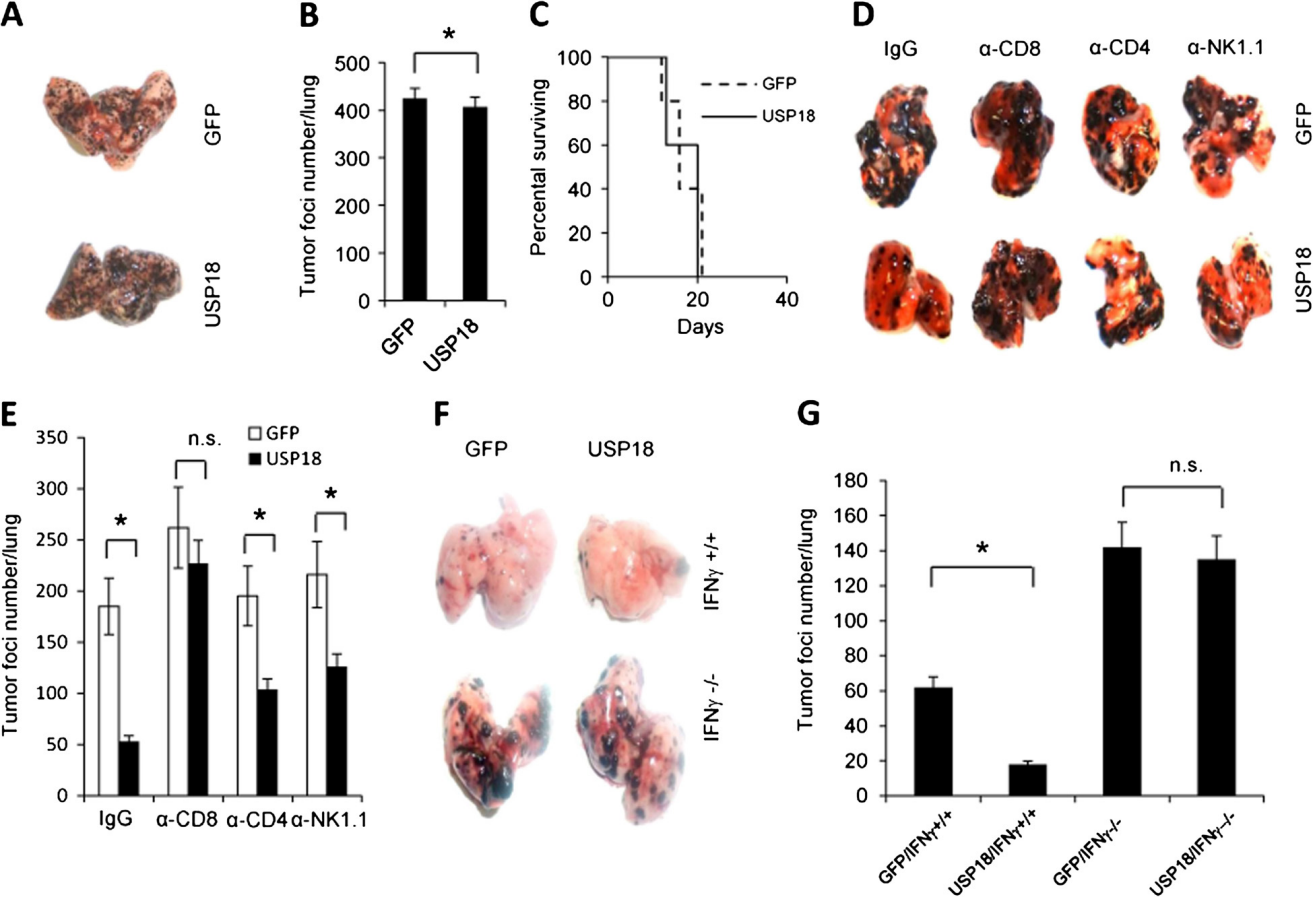
**USP18 function in tumor growth depends on the immune system.** 3 × 10^5^ B16-OVA-USP18 or B16-OVA-GFP tumor cells were intravenously inoculated into NSG mouse. The tumor burden **(A-B)** and mice survival rate **(C)** were analyzed. 3 × 10^5^ B16-OVA-USP18 or B16-OVA-GFP tumor cells were intravenously inoculated into C57BL/6 mice with or without depletion of CD8^+^ or CD4^+^ T cells, or NK1.1^+^ NK cells, and the tumor burden in the lung was monitored **(D-E)**. 3 × 10^5^ B16-OVA-GFP, or B16-OVA-USP18 tumor cells were intravenously inoculated into C57BL/6, or *Ifng*^-/-^ B6 mice. Tumor growth was monitored **(F-G)**.

To explore whether regulation of tumorigenesis by USP18 expression in tumor cells was due to host immune status, we intravenously inoculated B16-OVA-GFP or B16-OVA-USP18 tumor cells into NSG (NOD-SCID/IL2Rγ^-/-^) mice, which are deficient in T, B and NK cells. In contrast to immunocompetent mice, ectopic expression of USP18 did not inhibit tumorigenesis in NSG mice (Figure 3A and B), and no significant difference of survival in tumor-bearing mice was noted (Figure 3C). However, the tumor burden was higher in NSG mice compared with immunocompetent mice for both the B16-OVA-USP18 and B16-OVA-GFP inoculation groups, suggesting that lack of the immune system or immunosurveillance enabled the tumor cells to be more aggressive [15].

CD4^+^ and CD8^+^ T cells and NK cells are the principal IFN-γ-producing cells, and their anti-tumor activity depends on the robustness of IFN-γ production. To determine how tumor cell USP18 affects these cells and thus the immune response, we injected B16-OVA-GFP or B16-OVA-USP18 tumor cells into C57BL/6 mice that were depleted of CD4^+^ or CD8^+^ T cells or NK cells and monitored tumorigenesis. CD8^+^ T cell depletion significantly compromised the effect of USP18 expression on tumorigenesis (Figure 3D-E). However, CD4^+^ T cell or NK cell depletion was less important in USP18-mediated tumorigenesis (Figure 3D-E). To further investigate the function of USP18 in IFN-γ-mediated immune surveillance against tumorigenesis, B16-OVA-GFP and B16-OVA-USP18 cells were intravenously injected into IFN-γ-knockout or wide-type C57BL/6 mice. Tumor nodule formation was similar when B16-OVA-GFP and B16-OVA-USP18 cells were inoculated (intravenously) into IFN-γ-knockout (KO) mice (Figure 3F-G). These results showed that regulation of tumorigenesis by USP18 depended on IFN-γ signaling, most likely through the IFN-γ-mediated immune response.

### USP18 expression in tumor modulates immune cell population and phenotype

**Figure 4:**
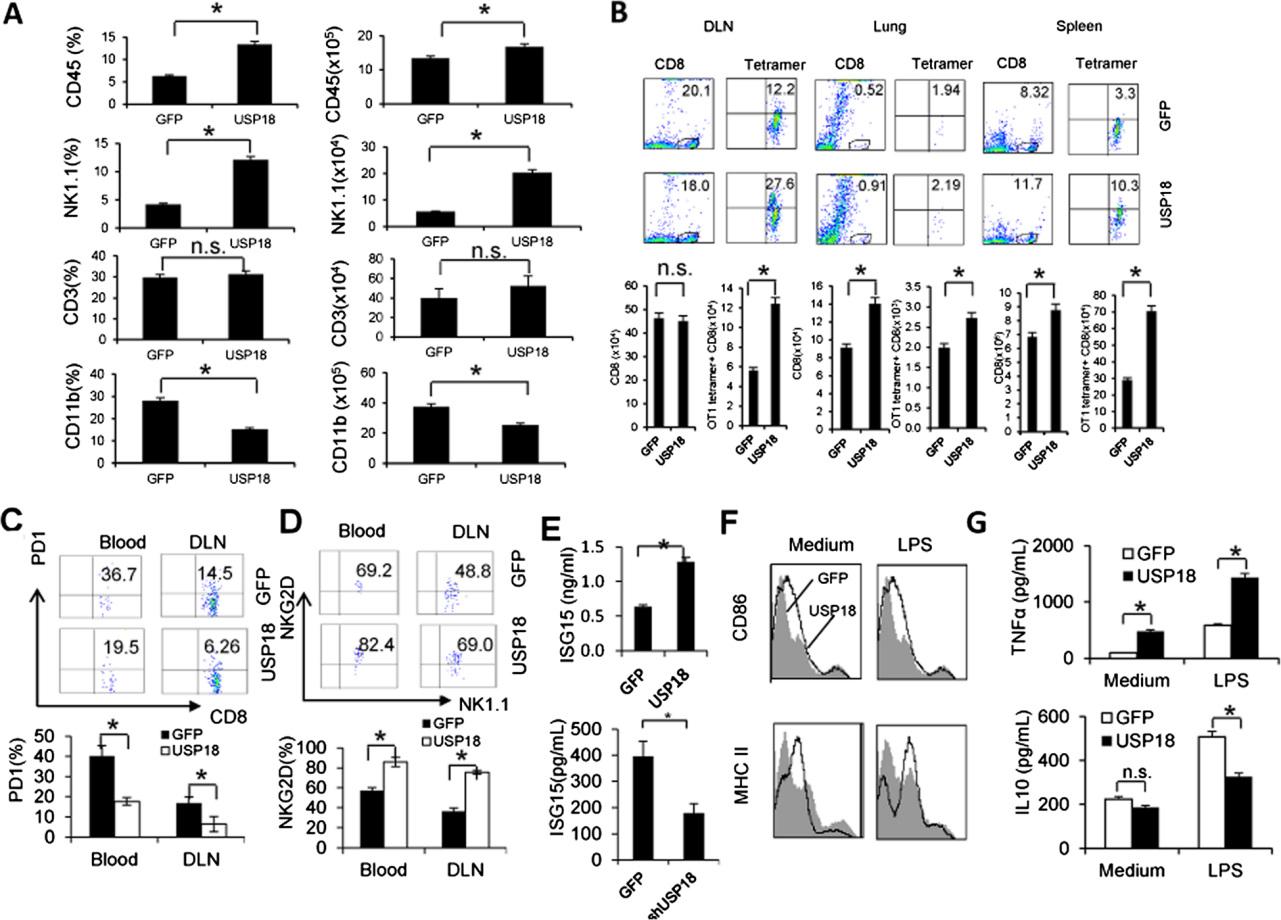
**USP18 expression in tumor modulates immune cell population and phenotype.** C57/BL6 mice received intravenous inoculation of 3 × 10^5^ B16-OVA-USP18 or B16-OVA-GFP tumor cells. 2 weeks later, T cell, CD11b^+^ myeloid cell and NK cell infiltration in the lung was analyzed by flow cytometry **(A)**. Antigen-specific OT-1 tetramer-positive CD8^+^ T cells in the lung draining lymph nodes (DLN), lung, and spleen were analyzed **(B)**. PD-1 expression on CD8^+^ T cells **(C)** was analyzed. NKG2D expression on NK cells in tumor microenvironment was monitored by flow cytometry **(D)**. ISG15 levels in B16-OVA-GFP, B16-OVA-USP18 or B16-OVA-shUSP18 tumor lysate was analyzed by ELISA **(E)**. CD11c^+^ dendritic cells were isolated from B16-OVA-GFP, or B16-OVA-USP18, and B16-OVA-shUSP18 lung melanoma and cultured in vitro with or without LPS. Dendritic cell maturation and cytokine were assayed by flow cytometry and ELISA, respectively **(F-G)**.

During tumor development, a variety of mechanisms inhibits the host immune response, including secretion of inhibitory cytokines and chemokines [16] and cell-cell contact suppression [17]. To explore USP18 function in immune suppression, we analyzed the immune status in the tumor microenvironment after USP18 overexpression in tumor cells (B16-OVA-USP18), which were then intravenously injected in C57BL/6 mice. CD45^+^ cell infiltration of the lung increased (Figure 4A). The percentage and the absolute number of NK cells also increased, but CD11b^+^ myeloid cell infiltration decreased (Figure 4A). Monitoring of SIINFEKL MHC class-I-restricted tetramer-positive CD8^+^ cells in the lung, spleen and draining lymph nodes revealed an increased antigen-specific CTL response in B16-OVA-USP18 tumor-bearing mice (Figure 4B).

Host immunosurveillance against tumorigenesis is not only determined by the potency of the antigen-specific immune response, but also by the persistence of immune effector cells [18]. Tumor cells have developed specific mechanisms to induce tolerance, inhibition, and exhaustion of immune effector cells, by altering the expression of PD-1 and KLRG1 on T cells [19,20]. Analysis of PD-1 expression in CD8^+^ T cells from B16-OVA-USP18 tumor-bearing mice showed a reduced PD-1 expression in the blood and draining lymph nodes compared to B16-OVA-GFP tumor-bearing mice (Figure 4C).

We speculated that USP18 expression in tumor cells might lead to secretion of soluble factors that also contribute to the immune activation. Cytokine array analysis of culture supernatant from USP18 overexpression and knockdown B16-OVA tumor cells did not show significant differences (Additional file 1: Figure S5A), but analysis of a chemokine panel showed that in B16-OVA-USP18 cells, increased secretion of chemokines related to NK cell migration was observed (Additional file 1: Figure S5B). In regard to NK cells, not only did the ratio and absolute number of infiltrating NK cells increased in B16-OVA-USP18 tumor-bearing mice (Figure 4A), but the activation status of the infiltrating NK cells also increased, as evidenced by detection of the activation receptor NKG2D (Figure 4D).

ISG15 is an IFN-induced gene that is regulated by USP18 [21]. In free form, ISG15 can activate NK cells and antigen presentation cells [22,23], and a recent study showed that human ISG15 deficiency leads to mycobacterial disease [24]. To detect ISG15 in the tumor microenvironment, tumor lysate was prepared from B16-OVA-GFP, B16-OVA-USP18 or B16-OVA-shUSP18 tumors, and ELISA or western blot analysis showed that the ISG15 level was higher in B16-OVA-USP18 tumor than in B16-OVA-GFP tumor, but lower in B16-OVA-shUSP18 tumor (Figure 4E, Additional file 1: Figure S6). We further investigated dendritic cell activity in the tumor microenvironment. CD11c^+^ dendritic cells were tolerized in the tumor stroma during tumor development [25]. Isolation of CD11c^+^ dendritic cells from B16-OVA-USP18 tumor bearing mice showed that these cells were activated instead of tolerized, because the expression of CD86 and MHC class-II was higher (Figure 4F), and more TNF-α but less IL-10 were secreted after re-stimulation in vitro as compared with B16-OVA-GFP tumor bearing mice (Figure 4G).

### USP18 expression inhibits immune suppression mediated by tumor cells

**Figure 5:**
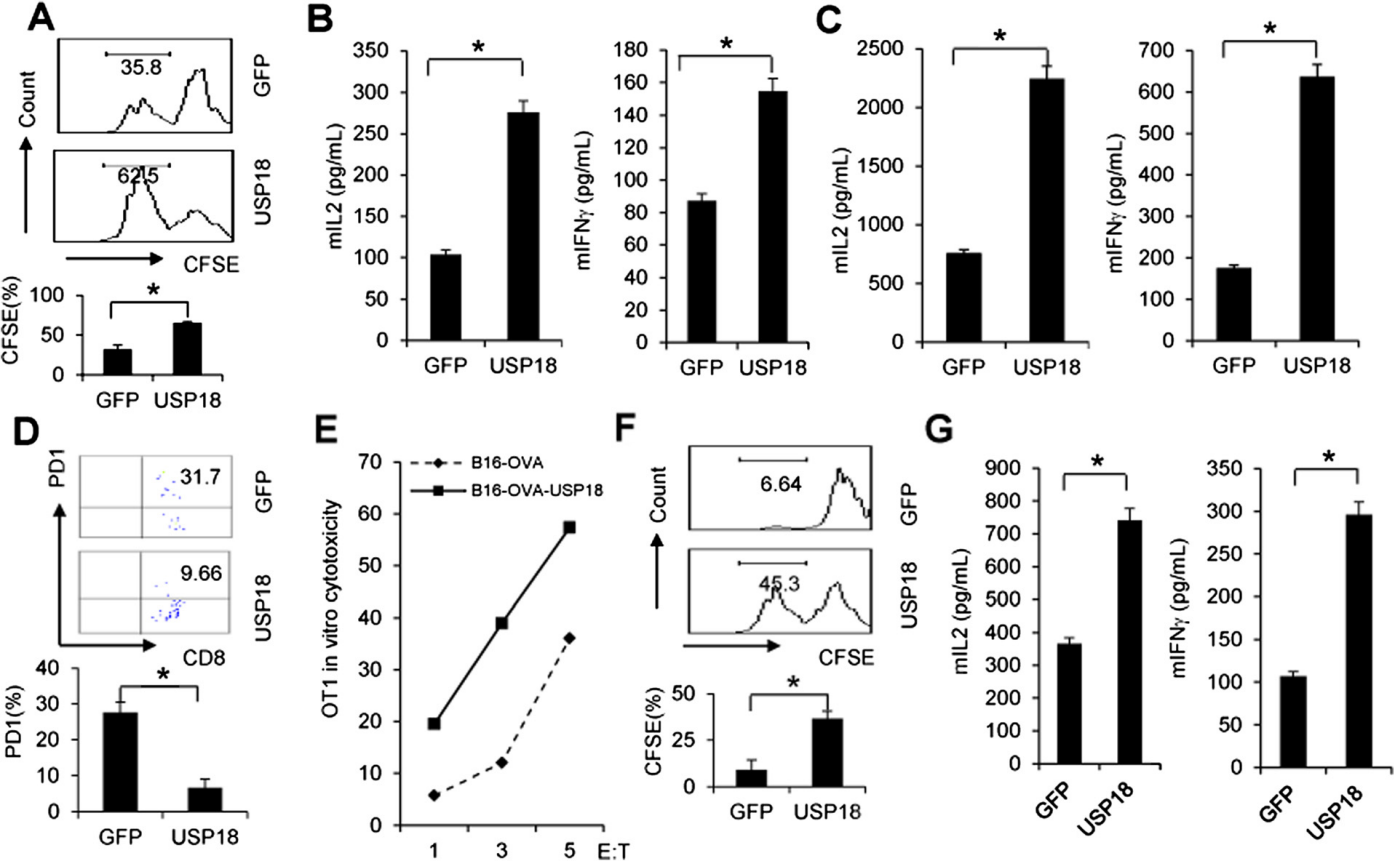
**Immune suppression by tumor cells is regulated by USP18 expression.** B16-OVA-GFP or B16-OVA-USP18 tumor cells without **(A-B)** or with IFN-γ sensitization (5 ng/ml) **(C)** were irradiated and cocultured with naïve OT-1 T cells. Cell proliferation **(A)** and IL-2 and IFN-γ secreteion **(B-C)** of the T cells were analyzed. PD-1 expression in cocultured OT-1 cells was monitored by flow cytometry **(D)**. The CTL activity of activated OT-1 cells against B16-OVA-GFP and B16-OVA-USP18 was analyzed **(E)**. B16-OVA-USP18 cells with or without IFN-γ (10 ng/ml) sensitization were irradiated and cocultured with naive OT-2 T cells for 72 hrs, and OT-2 T-cell proliferation **(F)** and cytokines IL-2 and IFN-γ secreteion **(G)** were analyzed.

As USP18 expression in tumor cells affects CD8^+^ T-cell function in vivo, B16-OVA-GFP or B16-OVA-USP18 cells were irradiated and then cocultured with OT-1 T cells in vitro to analyze T-cell activation. B16-OVA-USP18 tumor-cell coculture significantly increased OT-1 T-cell proliferation (Figure 5A), but also increased OT-1 T-cell secretion of IL-2 and IFN-γ (Figure 5B). Moreover, secretion of IL-2 and IFN-γ was even greater when OT-1 T cells were cocultured with IFN-γ-sensitized B16-OVA-USP18 tumor cells as compared with B16-OVA-GFP cells (Figure 5C). Activated T cells express inhibitory molecules, such as PD-1, to prevent over-activation [26]. We found that ectopic expression of USP18 in B16-OVA cells significantly inhibited PD-1 expression on cocultured OT-1 T cells (Figure 5D). This phenomenon was also seen in T cells from B16-OVA-USP18 cell inoculated mice (Figure 4C). Moreover, ectopic USP18 expression in B16-OVA tumor cells significantly increased the CTL activity of activated OT-1 cells in vitro (Figure 5E). CD4^+^ T cells also played a role in tumor immunosurveillance. Irradiated B16-OVA-USP18 cells stimulated OT-2 T cells to proliferate (Figure 5F) and secrete more IL-2 and IFN-γ (Figure 5G) after the coculture. Furthermore, overexpression or knockdown USP18 in B16-OVA cells did not affect OVA antigen expression level in tumor cells (Additional file 1: Figure S7), excluding the possibility that USP18 could regulate antigen expression.

### Inhibition USP18 activity in tumor cells compromises antigen-specific CTL activity

**Figure 6:**
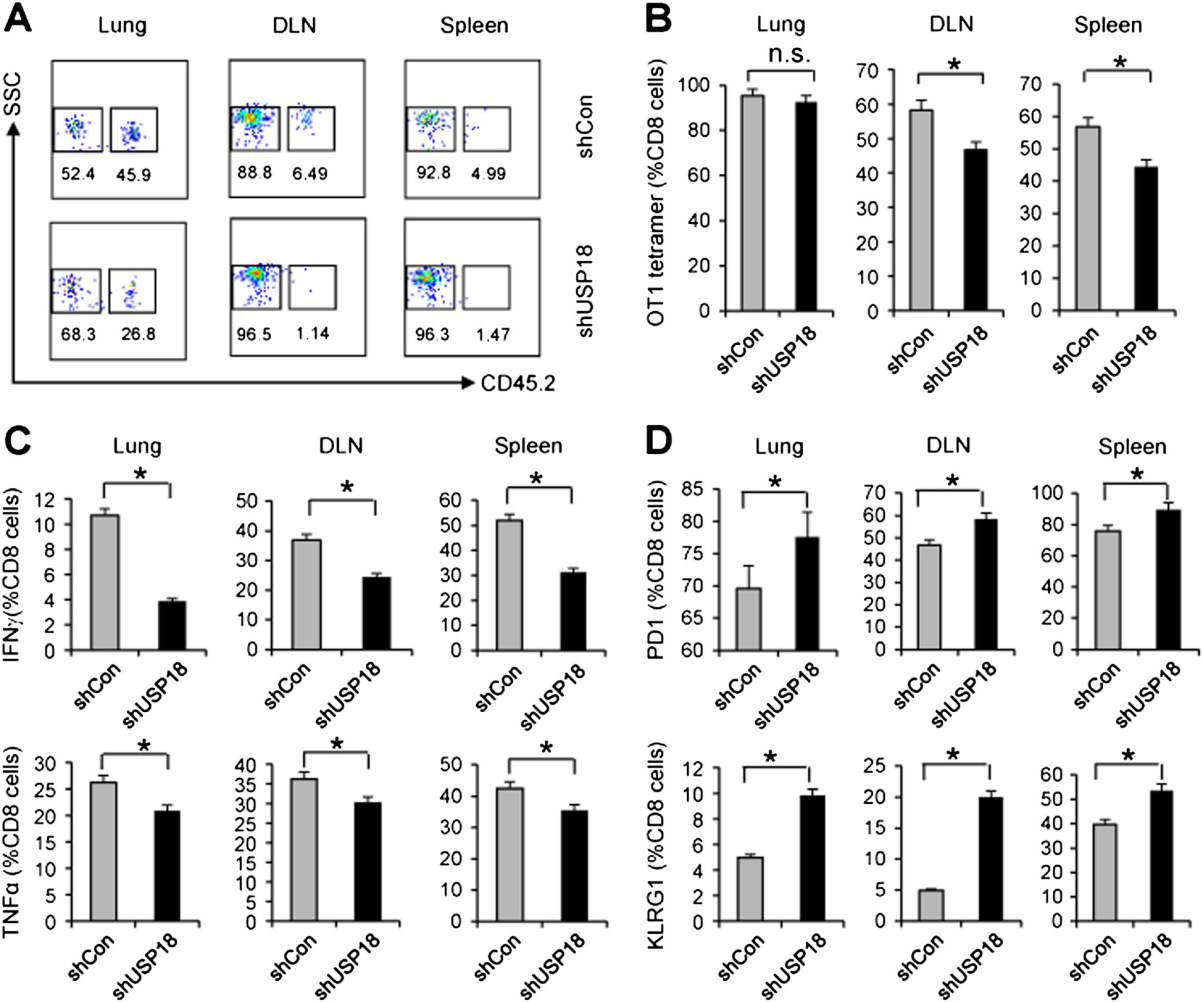
**Inhibition of USP18 activity in tumor cells compromises antigen-specific CTL activity.** CD45.1^+^ C57BL/6 mice received intravenous inoculation of 1 × 10^6^ B16-OVA-shCon and B16-OVA-shUSP18 tumor cells. At day 8, 5 × 10^6^ activated CD45.2^+^ OT-1 CTLs were intravenously injected into tumor-bearing mice. On day 10, the mice were sacrificed; CD45.2^+^ CTLs in lung, lung draining lymph nodes, and spleen were analyzed for OT-1 tetramer expression on CD8^+^ T cells (gating on CD8^+^ cell population) **(A-B)**, and IFN-γ and TNF-α **(C)**, and PD-1 and KLRG1 **(D)**.

As we found that endogenous IFN-γ signaling affected tumorigenesis through IFN-γ-induced USP18 expression in tumor cells and regulated immune-cell function in the tumor microenvironment, we wanted to explore whether USP18 expression in tumor cells affected exogenous IFN-γ-secreting CTL activity. Antigen-specific IFN-γ-secreting CTLs play an important role in antitumor immunity [27]. To analyze CTL activity in B16-OVA-shCon and B16-OVA-shUSP18 tumors, we intravenously injected 1 × 10^6^ B16-OVA-shCon cells or B16-OVA-shUSP18 cells into CD45.1^+^ C57BL/6 mice for 8 days, followed by adoptive transfer of 5 × 10^6^ activated OT-1 (CD45.2^+^) cells into tumor-bearing mice. Monitoring for persistence of CD45.2^+^ OT-1 cells in the lung, draining lymph nodes and spleen of tumor-bearing mice showed that OT-1 CTLs were rapidly exhausted in B16-OVA-shUSP18 tumor-bearing mice (Figure 6A). Tetramer (Figure 6B) and intracellular staining for IFN-γ and TNF-α (Figure 6C) demonstrated a loss of CTL activity in B16-OVA-shUSP18 tumor-bearing mice. Analysis of the T-cell exhaustion markers PD-1 and KLRG1 showed that the expression of these molecules was greater on OT-1 cells in B16-OVA-shUSP18 tumor-bearing mice than B16-OVA-shCon tumor-bearing mice (Figure 6D). These data indicated that USP18 expression in tumor cells regulated the IFN-γ-secreting activity and persistence of the CTLs in the tumor microenvironment.

### Ad-USP18 and CTL combination immunotherapy in subcutaneous B16 melanoma model

**Figure 7:**
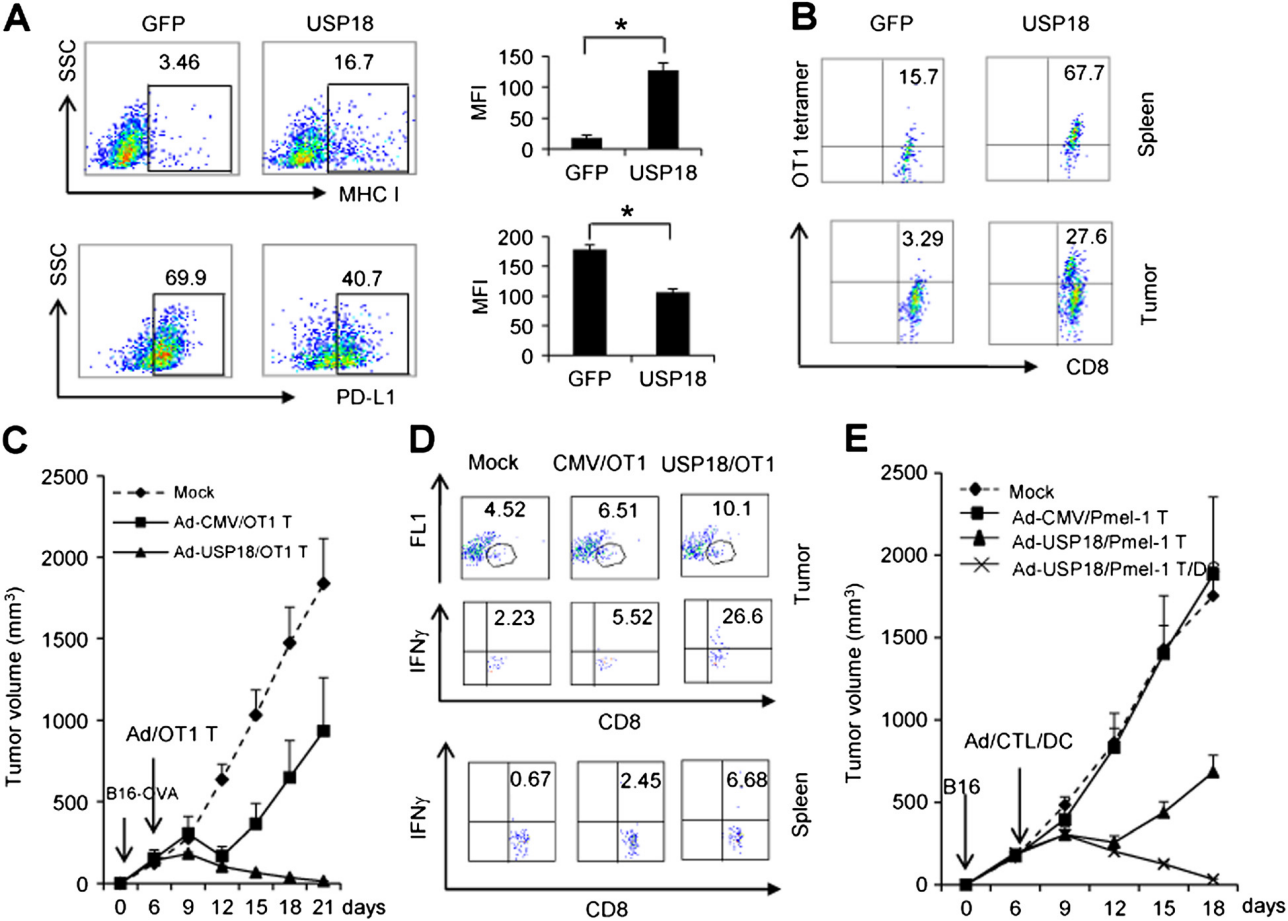
**Combination of Ad-USP18 and antigen-specific CTLs in subcutaneous B16 melanoma therapy.** 1 × 10^6^ B16-OVA-GFP or B16-OVA-USP18 cells were subcutaneously inoculated into C57BL/6 mice (n = 5). Tumor MHC class-I and PD-L1 expression was analyzed by flow cytometry **(A)**. Antigen-specific CTLs in the spleen and tumor were analyzed as OT-1 tetramer-positive CD8^+^ T cells **(B)**. 1 × 10^6^ B16-OVA cells were subcutaneously inoculated into CD45.1^+^ C57BL/6 mice. At day 5, tumor-bearing mice were treated with 5 × 10^6^ activated CD45.2^+^ OT-1 cells (intravenous) and intra-tumor injection of Ad-GFP or Ad-USP18 (10^10^ pfu in 50 μl). B16-OVA tumor growth was monitored. **(C)**. Effector CD45.2^+^ CD8^+^ T-cell persistence was measured 15 days after tumor inoculation **(D)**. 1 × 10^6^ B16 cells were subcutaneously inoculated into C57BL/6 mice. The tumor-bearing mice were treated with 5 × 10^6^ activated pmel-1^+^ CTLs (intravenous) along with intra-tumor injection of Ad-GFP or Ad-USP18 (10^10^ pfu in 50 μl) with or without dendritic cell vaccination. B16 tumor growth was monitored after CTL and Ad-USP18 treatment **(E)**.

Our study showed that USP18 expression in tumor cells regulated tumorigenesis and IFN-γ secretion by exogenous CTLs as part of the anti-tumor immune response. Next, we investigated the possibility that use of USP18 could enhance CTL activity against subcutaneous B16 melanoma. After B16-OVA-GFP, B16-OVA-USP18, or B16-OVA-shUSP18 tumor cells were subcutaneously inoculated into C57BL/6 mice, tumor growth in mice receiving B16-OVA-USP18 cells was significantly inhibited, whereas tumor growth was promoted in those receiving B16-OVA-shUSP18 tumor cells (Figure 2G). Next, we determined whether MHC class-I and PD-L1 expression differed in subcutaneous B16-OVA-USP18 tumor from that of B6-OVA-GFP tumor. Results showed that ectopic expression of USP18 increased MHC class-I and reduced PD-L1 expression (Figure 7A). As MHC class-I and PD-L1 expression on tumor cells significantly affected host immunosurveillance, we also detected greater OT-1 tetramer-positive CD8^+^ CTL infiltration in B16-OVA-USP18 tumor than those in B16-OVA-GFP tumor (Figure 7B). Moreover, more effector T cells were activated in B16-OVA-USP18 tumor and spleen compared with B16-OVA-GFP tumor and spleen (Additional file 1: Figure S6A and B). However, there was no difference in infiltrating regulatory T cells between B16-OVA-USP18 and B16-OVA-GFP subcutaneous tumor (Additional file 1: Figure S6C).Next we explored whether delivery of USP18 into the tumor microenvironment enhances CTL activity against subcutaneous B16-OVA tumor. A subcutaneous B16-OVA tumor model was established in C57BL/6 mice. At day 5, activated OT-1 CTLs were adoptively transferred to the mice, together with intra-tumor injection of adenovirus vector expressing USP18 (Ad-USP18). Adoptive transfer of OT-1 CTLs induced partial tumor shrinkage (Figure 7C). Ad-USP18 injection increased OT-1 CTL activity and tumor shrinkage (Figure 7C). Detailed analysis of the immune status in tumor-bearing mice showed that Ad-USP18 significantly increased the duration of CTL persistence in the spleen and tumor sites of tumor-bearing mice (Figure 7D).

As tumor antigens are weakly immunogenic, dendritic cell vaccination and IL-2 treatment are usually used to increase CTL activity clinically [28]. Using our B16 tumor model, we co-administered pmel-1 CTLs, Ad-USP18, IL-2 and dendritic cells into tumor. Injection of Ad-USP18 significantly increased pmel-1 CTL activity and caused tumor shrinkage (Figure 7E).

## Discussion

The novel finding in this study is the discovery that the ubiquitin-specific peptidase USP18 can be induced by IFN-γ in tumor cells and plays important roles in inhibiting tumorigenesis and antitumor immunity. We found that USP18 is expressed in tumor cells after IFN-γ signaling stimulation. Ectopic expression of USP18 in tumor cells suppressed tumorigenesis and antitumor immune response whereas inhibition of USP18 expression promoted tumorigenesis and immunosurveillance. Mechanistically, USP18 expression in tumor cells modulated immune-cell population and phenotype in tumor microenvironment. Moreover, USP18 controlled exogenous IFN-γ producing antigen-specific CTL persistence in antitumor immunity.

Many signaling pathways are involved in the regulation of tumorigenesis and immunosurveillance. Type-I and-II IFNs are central in a broad signaling network that regulates tumor-cell proliferation and apoptosis, and modifies both the anti-viral and antitumor immune responses. Type-I IFN signaling has been recently reported to be involved in tumor metastasis, e.g., genes suppressed in bone metastasis are targets of IFN regulatory factor 7 (IRF7) [29]. USP18 expression in tumor cells, such as human sarcoma 2fTGH cells, is not only induced by IFNγ timulation [30]. Its expression or mutation has also been reported to be disregulated during tumorigenesis or treatment [31,32]. Combination with other markers, USP18 can be used to predict the cancer patients survival [33]. We found that USP18, which is induced by IFN-α/-β in response to viral infection, is also expressed in tumor cells after endogenous and exogenous type-II IFN-γ signaling activation.

The mechanism underlying USP18 function in tumorigenesis and antitumor immunity involves activation of tumor immunosurveillance and alteration of the tumor microenvironment. That endogenous IFN signaling through USP18 controls tumorigenesis is evidenced by tumor growth in the *Ifng*^*-/-*^ mice after inoculation with B16-OVA-USP18 or B16-OVA-shUSP18 tumor cells. However, it should be noted that in this study, USP18 expression in tumor cells not only affected tumor cell activity, but also regulated immune cells in tumor microenvironment in that tumor cell USP18 expression also activated CD11c^+^ dendritic cells residing in the tumor. Besides our report, USP18 has been reported to directly maintain oncogene stability such as the chimeric, dominant-negative-acting transcription factor promyelocytic leukemia gene (PML)/RARa during acute promyelocytic leukemia development [34]. USP18 also stabilize cyclin D of tumor cells to inhibit apoptosis [35]. All these reports suggested that USP18 may have both anti- and pro-oncogenic activity depending on the target cells in the tumor environment.

Immunotherapy has become an important approach to increase the efficacy of chemotherapy or radiotherapy [36]. Clinical trials have shown that after immunotherapy, some melanoma and prostate cancer regressed [37]. Immunotherapy effectiveness is compromised because negative regulation mechanism in the immune system [10,38]. Our study showed that USP18 expression in tumor cells significantly suppressed the expression of molecules responsible for inhibiting CTL activity during immunotherapy. Moreover enhancing USP18 expression in primary tumors by administration of adenovirus vector encoding USP18 significantly increased antigen-specific CTL activity.

Gene therapy against tumor utilizes the delivery of DNA into cells, which can be accomplished by a variety of methods. Adenovirus vector is the efficient system to deliver target DNA therapy in vivo [39], as it has higher infection efficiency on both tumor cells and immune cells. In our study, delivering USP18 into solid tumor could be accomplished by intra-tumor injection of adenovirus containing USP18 cDNA. Tumor microenvironment contains a variety of other cells, such as immune cells and stromal cells, and USP18 could regulate immune cells [12]. Manipulation of USP18 expression not only induces possible anti-apoptotic activity in some cancers [35], but also change the tumor cell sensitivity to immunotherapy. Moreover, overexpression of USP18 in tumor cells may induce the immunesuppression activity of MDSC in tumor environment. Further studies will be performed to investigate the influence of Ad-USP18 on immune cells in tumor microenvironment.

In conclusion, we found that IFN-γ signaling induces intrinsic expression of USP18 in tumor cells that not only affects tumorigenesis, but also may be useful in regulating immunotherapy efficacy.

## Competing interests

The authors declare that they have no competing financial interests.

## Authors’ contributions

The author(s) have made the following declarations about their contributions: Conceived and designed the experiment: BH, QY. Performed the experiments: BH, HL, MZ, YL. Analyzed the data: BH, HL, MZ, YZ, JQ. Wrote the paper: BH, QY. Supervised the work: QY. All authors read and approved the final manuscript.

## Supplementary Material

Additional file 1: Figure S1 MHC class-I expression in B16 tumor cells with or without IFN-γ signaling. **Figure S2.** Proliferation and apoptosis of B16-OVA cells after overexpression or knockdown of USP18. **Figure S3.** Overexpression or knockdown USP18 in B16-OVA cells affects tumor growth. **Figure S4.** Adhesion, invasion and migration activity of B16 cells after overexpression or knockdown of USP18 expression. **Figure S5.** Overexpression of USP18 in B16 cells does not affect cytokine secretion in vitro but does affect chemokine production in the tumor microenvironment. **Figure S6.** ISG15 levels in B16-OVA-GFP, B16-OVA-USP18 or B16-OVA-shUSP18 tumor lysate. **Figure S7.** OVA expression in B16-OVA-GFP, B16-OVA-USP18 and B16-OVA-shUSP18 tumor cells. **Figure S8.** Overexpressionof USP18 in B16-OVA cells affects subcutaneous tumorigenesis. Click here for file
