# Supplementary material for: USP18 is crucial for IFN-γ-mediated inhibition of B16 melanoma tumorigenesis and antitumor immunity
Source: Mol Cancer. 2014 May 31;13:132. doi: 10.1186/1476-4598-13-132 (PMC4057584; doi:10.1186/1476-4598-13-132)
Supplement: Additional file 1: Figure S1 — MHC class-I expression in B16 tumor cells with or without IFN-γ signaling. Figure S2. Proliferation and apoptosis of B16-OVA cells after overexpression or knockdown of USP18. Figure S3. Overexpression or knockdown USP18 in B16-OVA cells affects tumor growth. Figure S4. Adhesion, invasion and migration activity of B16 cells after overexpression or knockdown of USP18 expression. Figure S5. Overexpression of USP18 in B16 cells does not affect cytokine secretion in vitro but does affect chemokine production in the tumor microenvironment. Figure S6. ISG15 levels in B16-OVA-GFP, B16-OVA-USP18 or B16-OVA-shUSP18 tumor lysate. Figure S7. OVA expression in B16-OVA-GFP, B16-OVA-USP18 and B16-OVA-shUSP18 tumor cells. Figure S8. Overexpressionof USP18 in B16-OVA cells affects subcutaneous tumorigenesis. [file 1476-4598-13-132-S1.pdf]

Supplemental figure legends

Supplemental Figure 1. MHC class-I expression in B16 tumor cells with or without IFN-γ signaling. MHC class-I expression on B16 cells after IFN signaling sensitization in vitro (**A**) or IFN signaling deficiency in vivo (**B**). MHC class-I expression on B16 cells after IFN signaling sensitization in vitro (**A**) or IFN signaling deficiency in vivo (**B**).

Supplemental Figure 2. Proliferation and apoptosis of B16-OVA cells after overexpression or knockdown of USP18. USP18 expression in B16-OVA cells was overexpressed or knocked down using lentivirus vector and confirmed by Western blot assay (**A** & **B**). Exponentially growing B16-OVA tumor cells stably expressing shUSP18 (B16-OVA-shUSP18) were pulsed with 10 μM BrdU and cell proliferation was analyzed by flow cytometry (**C**). The apoptosis status of B16-OVA-shUSP8 cells was analyzed by flow cytometry staining for Annexin/PI (**D**).

Supplemental Figure 3. Overexpression or knockdown USP18 in B16-OVA cells affects tumor growth. 3×10^5 B16-OVA-GFP, B16-OVA-USP18 (2) or B16-OVA-shUSP18(2) tumor cells were intravenously inoculated into C57BL/6 mice. Tumor growth was monitored 3weeks after inoculation. B16-OVA-USP18 (2) and B16-OVA-shUSP18(2) cells lines are from different clone or shRNA.

Supplemental Figure 4. Adhesion, invasion and migration activity of B16 cells after overexpression or knockdown of USP18 expression. The ability of B16-OVA,

B16-OVA-USP18 and B16-OVA-shUSP18 tumor cells adhering to fibronectin-coated plates were analyzed (**A**). The invasion and migration ability of B16-OVA, B16-OVA-USP18 and B16-OVA-shUSP18 cells was analyzed in transwell plates coated with fibronectin (**B**) or matrigel (**C**).

Supplemental Figure 5. Overexpression of USP18 in B16 cells does not affect cytokine secretion in vitro but does affect chemokine production in the tumor microenvironment. Cytokine and chemokine secretion from culture supernatant of B16-OVA, B16-OVA-USP18 and B16-shUSP18 tumor cells was analyzed by cytokine array (**A**). Chemokine mRNA level in B16-OVA-GFP and B16-OVA-USP18 tumor microenvironment were analyzed by qRT-PCR (**B**).

Supplemental Figure 6. ISG15 levels in B16-OVA-GFP , B16-OVA-USP18 or B16-OVA-shUSP18 tumor lysate. ISG15 levels in B16-OVA-GFP, B16-OVA-USP18 or B16-OVA-shUSP18 tumor lysate was analyzed by Western blot assay.

Supplemental Figure 7. OVA expression in B16-OVA-GFP, B16-OVA-USP18 and B16-OVA-shUSP18 tumor cells. Western blot assay of OVA protein expression in B16-OVA tumor cells with overexpression or knockdown of USP18.

Supplemental Figure 8. Overexpression of USP18 in B16-OVA cells affects subcutaneous tumorigenesis. C57BL/6 mice received subcutaneous inoculation of 1×10^6 B16-OVA-GFP and B16-OVA-USP18 cells. Effector ($CD44^+$) T cells in tumor (**A**) and spleen (**B**) was analyzed. $CD4^+Foxp3^+$ regulatory T cells located in spleen

were analyzed by flow cytometry (**C**).

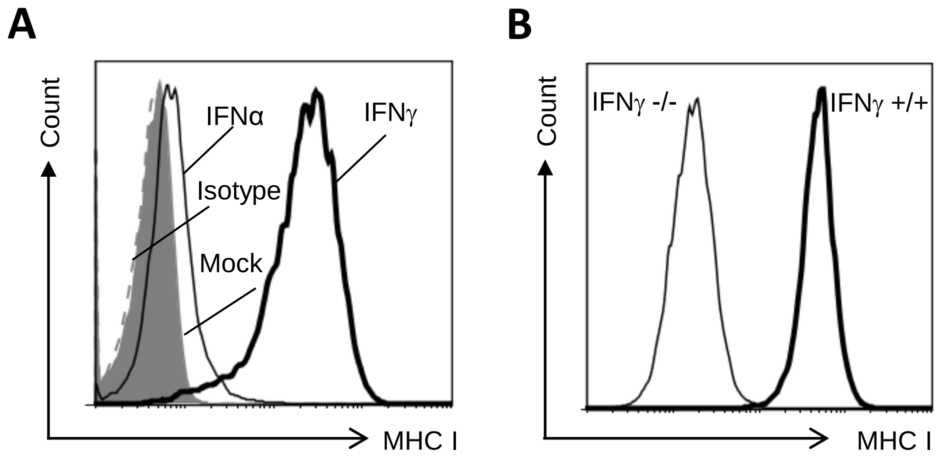

sFigure 1.

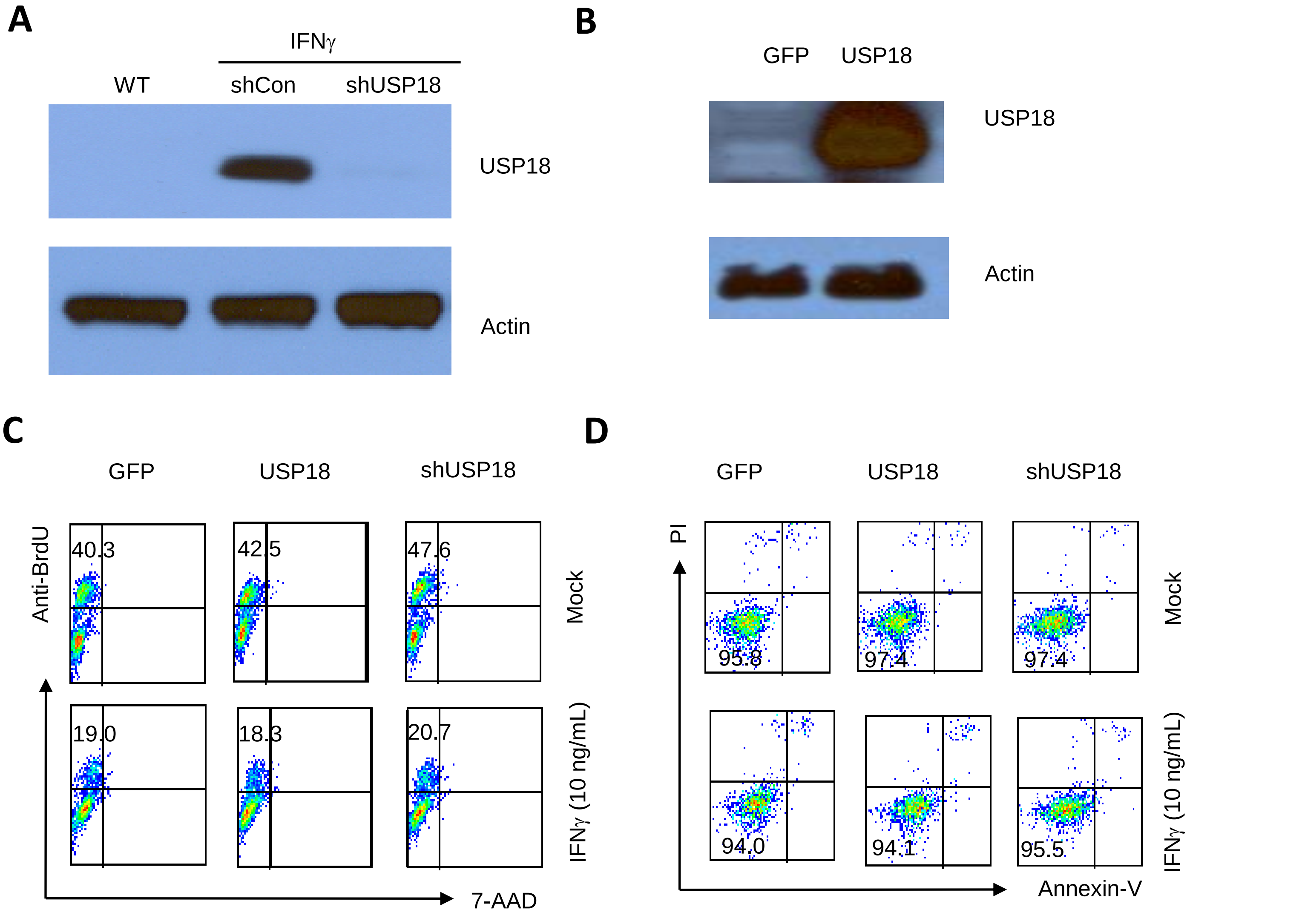
A
IFNγ
WT
shCon
shUSP18
USP18
Actin
B
GFP
USP18
USP18
Actin
C
GFP
USP18
shUSP18
Anti-BrdU
40.3
42.5
47.6
Mock
19.0
18.3
20.7
IFNγ (10 ng/mL)
7-AAD
D
GFP
USP18
shUSP18
PI
95.8
97.4
97.4
Mock
94.0
94.1
95.5
IFNγ (10 ng/mL)
Annexin-V

sFigure 2.

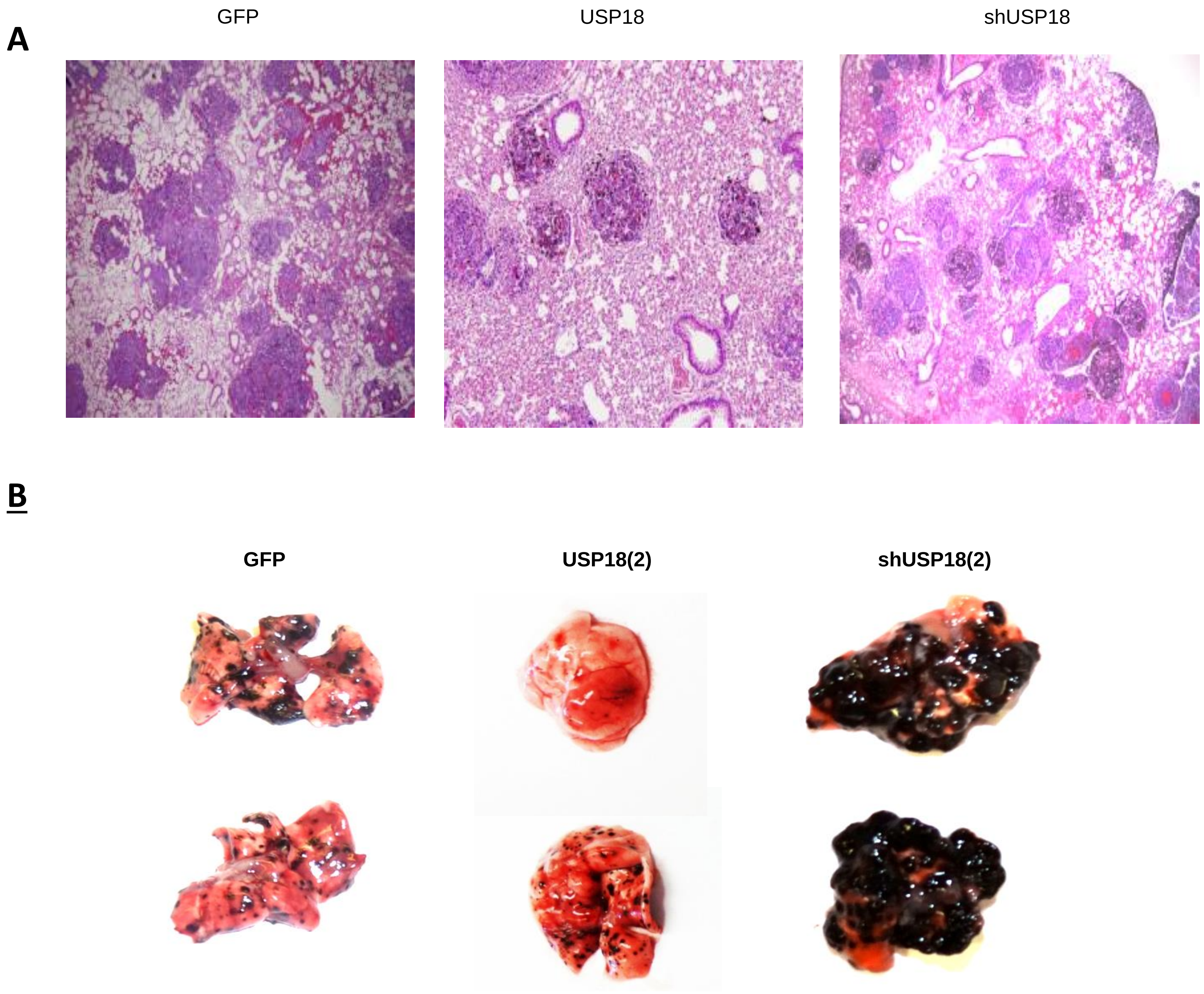

sFigure 3.

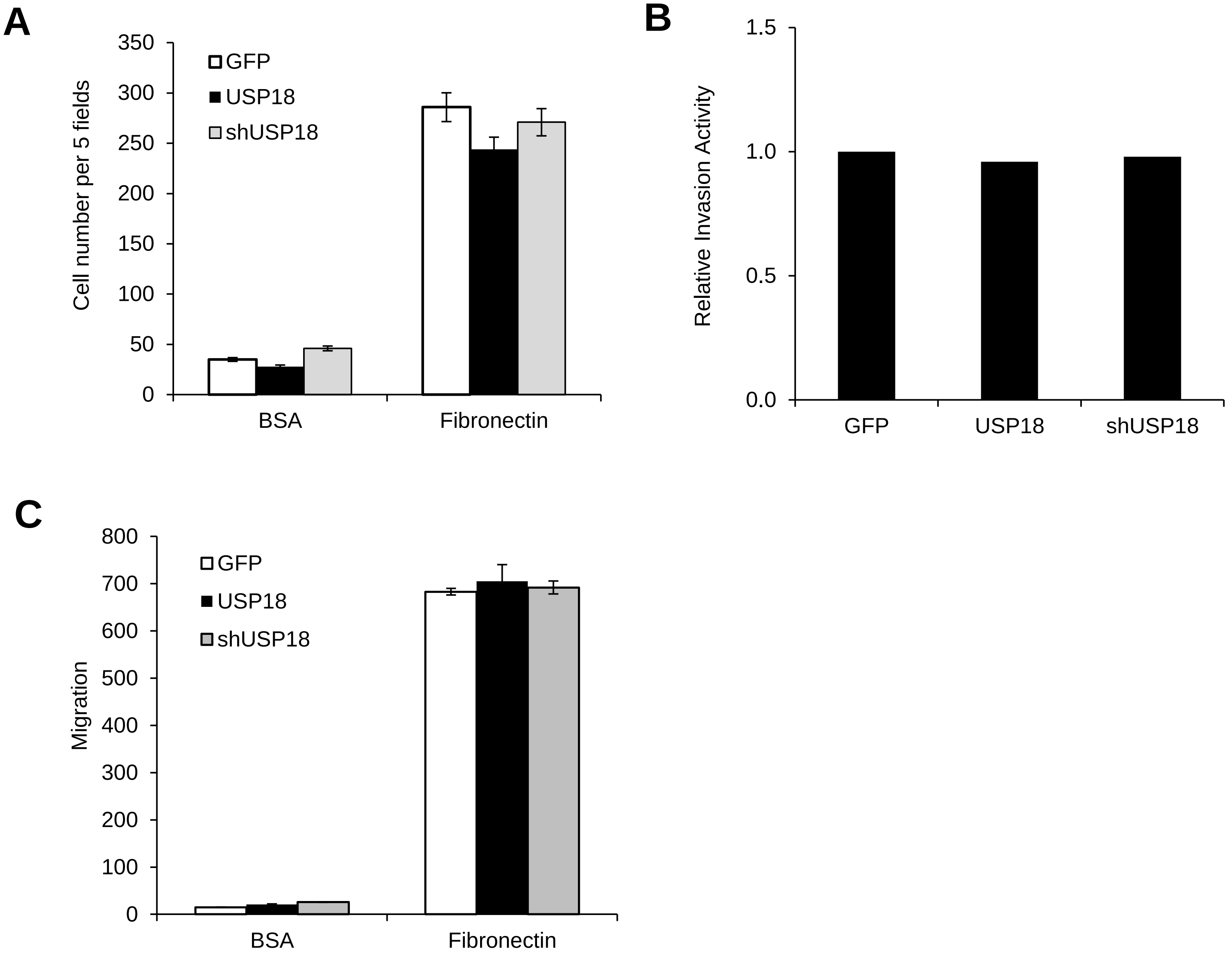

A
350
300
250
200
150
100
50
0
Cell number per 5 fields
GFP
USP18
shUSP18
BSA
Fibronectin
B
1.5
1.0
0.5
0.0
Relative Invasion Activity
GFP
USP18
shUSP18
C
800
700
600
500
400
300
200
100
0
Migration
GFP
USP18
shUSP18
BSA
Fibronectin

sFigure 4.

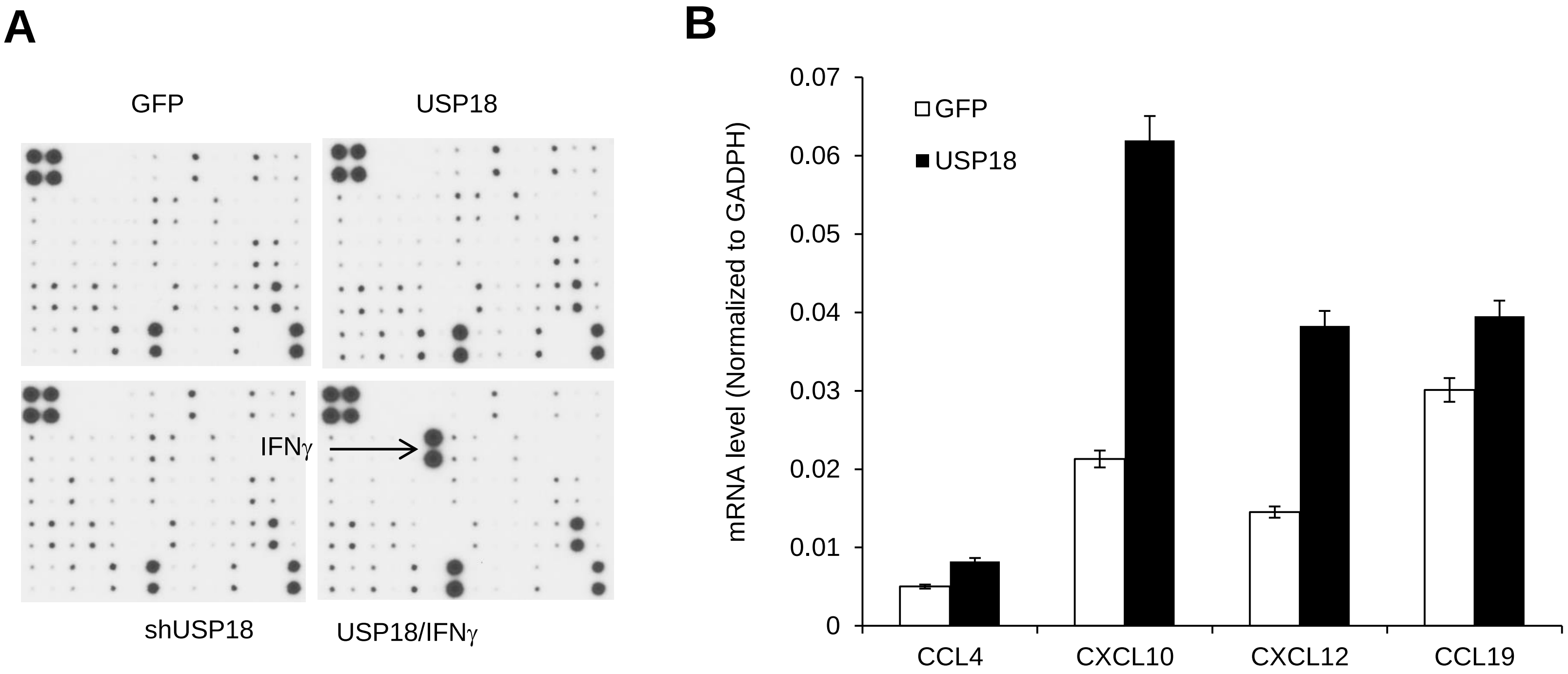
A
GFP
USP18
IFNγ
shUSP18
USP18/IFNγ
B
0.07
0.06
0.05
0.04
0.03
0.02
0.01
0
GFP
USP18
mRNA level (Normalized to GADPH)
CCL4
CXCL10
CXCL12
CCL19

sFigure 5.

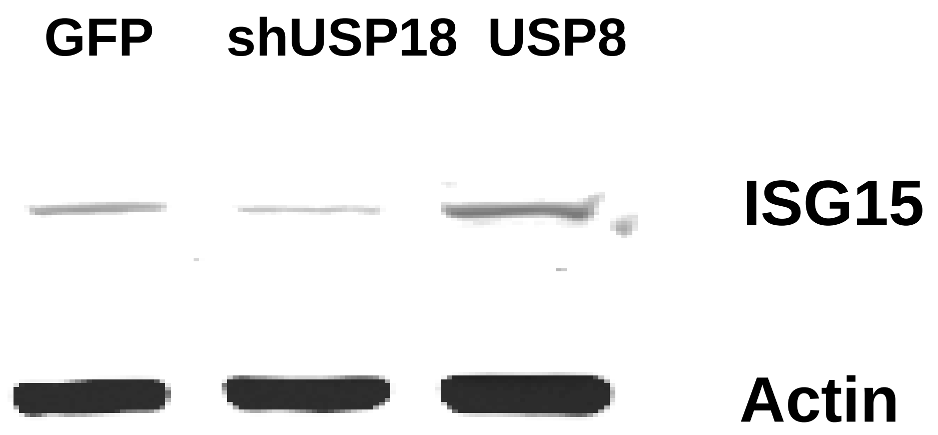

sFigure 6.

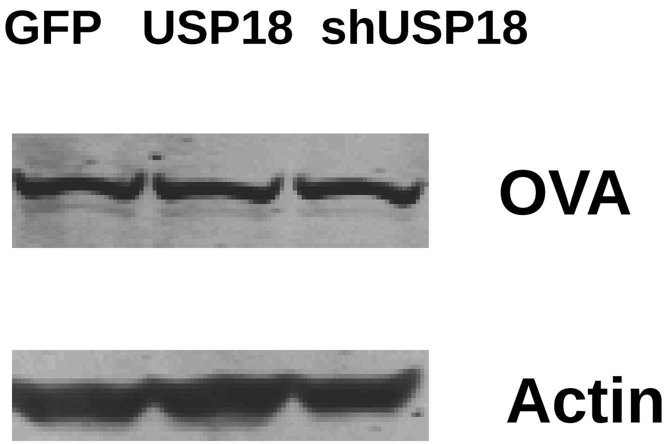

sFigure 7.

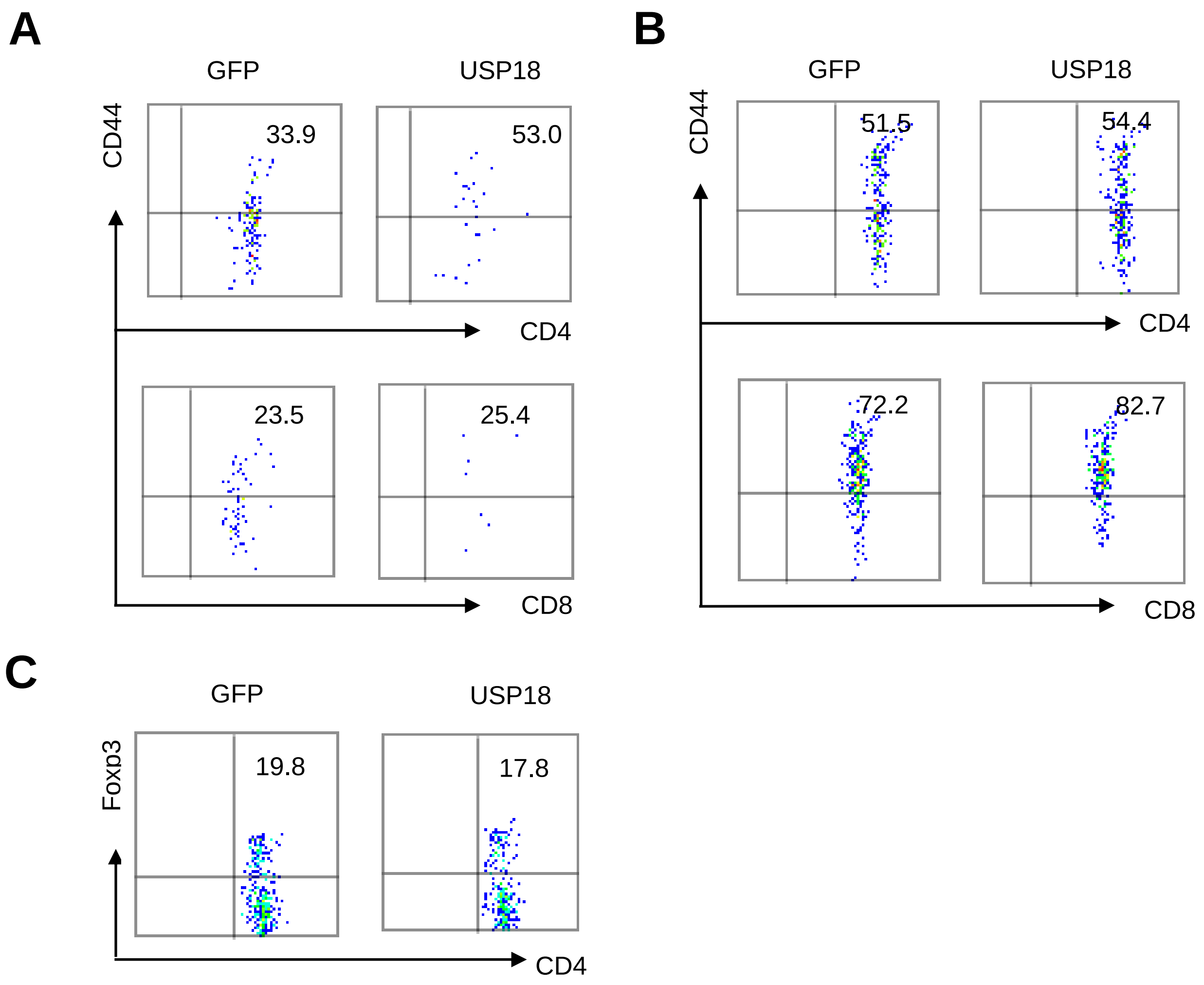
A
GFP
USP18
CD44
33.9
53.0
CD4
23.5
25.4
CD8
B
GFP
USP18
CD44
51.5
54.4
CD4
72.2
82.7
CD8
C
GFP
USP18
Foxp3
19.8
17.8
CD4

sFigure 8.
